# Mitochondria as the central regulator of cell death in bronchopulmonary dysplasia

**DOI:** 10.3389/fphys.2025.1685526

**Published:** 2025-09-15

**Authors:** Fan Zhang, Laishuan Wang, Yahui Zhou

**Affiliations:** ^1^ Neonatal Department, Children’s Hospital Affiliated to Jiangnan University (Wuxi Children’s Hospital), Wuxi, Jiangsu, China; ^2^ Neonatal Medical Center, National Health Commission Key Laboratory of Neonatal Diseases, Children’s Hospital of Fudan University, Shanghai, China

**Keywords:** bronchopulmonary dysplasia, mitochondria, oxidative stress, inflammation, apoptosis, autophagy, cell death

## Abstract

Bronchopulmonary dysplasia (BPD) remains a major chronic respiratory condition affecting preterm infants, characterized by impaired alveolar and vascular development. While the roles of oxidative stress and inflammation are recognized, this review provides a novel synthesis by positioning mitochondrial dysfunction as the central hub integrating these injurious processes with the activation of diverse cell death pathways in BPD pathogenesis. We critically explore how mitochondrial damage, driven by hyperoxia and inflammation, not only amplifies cellular injury but also orchestrates specific modes of programmed cell death, particularly apoptosis, pyroptosis, necroptosis, autophagy, ferroptosis, and the newly delineated cuproptosis. A key contribution is elucidating the crosstalk between these pathways and their collective impact on alveolar simplification and vascular dysregulation. Furthermore, we discuss the translational implications of targeting mitochondrial quality control and death pathways, proposing novel biomarkers and therapeutic strategies aimed at mitigating BPD progression. This review thus offers a unified mitochondrial-centric perspective, moving beyond descriptive mechanism to provide a conceptual framework for understanding BPD pathobiology and advancing targeted interventions.

## 1 Introduction

Bronchopulmonary dysplasia (BPD) represents a chronic lung disease of prematurity, resulting from multifactorial perinatal insults that disrupt the development of immature lungs (Schmidt and Ramamoorthy, 2022). Its radiographic features include extensive streaks and hyperinflation, and it remains the most common complication among preterm infants, affecting up to 45% of those born at ≤ 29 weeks’ gestation (Thébaud et al., 2019). The classic pathological hallmark of fibrocystic changes in late preterm infants has shifted toward a modern phenotype characterized by impaired alveolar development and dysregulated vascularization in extremely preterm infants (Stoecklin et al., 2019). Pathologically, BPD involves alveolar-capillary developmental arrest, simplified alveolar structures, interstitial fibrosis, and abnormal pulmonary vascular remodeling (Gilfillan et al., 2021). Infants born during the stage of canalicular lung development, when alveolar differentiation and airway formation occur, are at highest risk (Rodríguez-Castillo et al., 2018). The pathogenesis of BPD involves complex interactions between genetic susceptibility, environmental exposures (e.g., mechanical ventilation, hyperoxia), and oxidative stress (OS) (Shukla and Ambalavanan, 2021). Despite advances in neonatal care, including antenatal corticosteroids and lung-protective ventilation, a significant proportion of affected infants progress to severe BPD, with long-term sequelae such as pulmonary hypertension and emphysema (Wang and Tsao, 2020; Dini et al., 2024). These sequelae contribute to chronic respiratory disability and pose significant clinical and economic burdens. Notably, while modern neonatal interventions have dramatically improved the survival of extremely preterm infants, the persistent high incidence of BPD and its poor long-term outcomes underscore an unresolved paradox: life-saving therapies may inadvertently perpetuate lung injury. This pressing dilemma calls for further research to reconcile improved survival with enhanced pulmonary outcomes, ultimately mitigating the lifelong health consequences of BPD. In-depth exploration of the pathological mechanism of BPD may provide a new theoretical basis for the long-term prognosis of BPD.

Mitochondria, often termed the cell’s “powerhouses”, are double-membrane-bound organelles that serve as the primary site of oxidative phosphorylation (Eldeeb et al., 2022). Beyond energy production, they play pivotal roles in cellular homeostasis, reactive oxygen species (ROS) scavenging, and intrinsic apoptotic signaling (Xuefei et al., 2021). In preterm infants with BPD, mitochondrial immaturity increases susceptibility to postnatal insults such as hyperoxia, mechanical ventilation, and infection. These factors induce OS, causing mitochondrial dysfunction characterized by disrupted ATP production and compromised antioxidant defenses (Zhao et al., 2024). Excessive ROS directly damage alveolar type II epithelial (AT2) cells as well as pulmonary endothelial cells, leading to impaired surfactant production, arrested alveolarization, and dysregulated angiogenesis (Wang and Dong, 2018; Bonadies et al., 2020). Cellular damage is further exacerbated, and the progression of BPD is accelerated through the activation of mitochondrial-dependent cell death pathways, including apoptosis, autophagy, and ferroptosis (Deng et al., 2023; Wang et al., 2023; Ruchko et al., 2005). This “insult-mitochondria-cell death” axis represents a core pathological mechanism in BPD progression.

For this reason, our review centers on the pathological mechanisms of mitochondrial dysfunction and mitochondrial-dependent cell death in relation to BPD. It aims to identify potential therapeutic targets and provide new avenues for the intervention of BPD.

## 2 BPD and mitochondrial dysfunction

**FIGURE 1:**
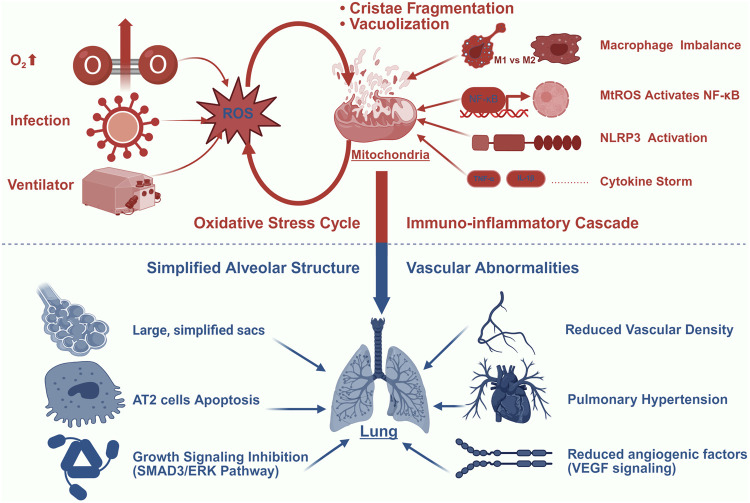
BPD and Mitochondrial Dysfunction. This figure illustrates the central role of mitochondrial damage in driving BPD progression through four interconnected mechanisms: (1) Oxidative Stress Amplification: Hyperoxia (O_2_↑), mechanical ventilation, and infection generate excessive ROS, inducing mitochondrial structural damage (cristae fragmentation and vacuolization) that further amplifies mtROS production; (2) Immuno-inflammatory Cascade: mtROS activates NF-κB signaling and NLRP3 inflammasome, promoting macrophage polarization imbalance (M1/M2 shift) and cytokine storm (elevated TNF-α/IL-1β); (3) Alveolar Simplification: Persistent stress induces AT2 cell apoptosis and inhibits growth signaling (SMAD3/ERK pathways), resulting in impaired alveolar septation; (4) Vascular Dysregulation: Mitochondrial dysfunction reduces VEGF signaling and angiogenic factors, leading to diminished vascular density and pulmonary hypertension. Color key: Red indicates oxidative/inflammatory processes; blue indicates structural abnormalities.

The pathogenesis of BPD is mediated through mitochondrial dysfunction, a process primarily driven by the interaction of OS, immune dysregulation, and developmental arrest. OS plays a key role as a major trigger of mitochondrial damage, initiating a cascade of cellular injury that drives the progression of BPD. The underdeveloped lungs of preterm infants are especially susceptible to redox imbalances due to underdeveloped antioxidant defenses, creating an environment in which exogenous insults, such as hyperoxia and mechanical ventilation induce irreversible mitochondrial damage. These insults not only disrupt pulmonary vascular endothelial metabolism and alveolar epithelial homeostasis but also enhance inflammatory responses, collectively impairing lung development (Figure 1).

### 2.1 OS

OS and mitochondrial dysfunction form a mutually reinforcing vicious cycle in the pathogenesis of BPD. Various external stimuli, such as iatrogenic hyperoxic environments, can trigger the production of excessive ROS and reactive nitrogen species in the body, which lead to tissue damage through multiple pathways. These oxidants not only induce oxidative damage to lipids, proteins, and DNA, particularly mitochondrial DNA (mtDNA), a genetic material highly sensitive to OS (Kandasamy et al., 2023), but also impair the function of critical biomolecules via modifications such as S-nitrosylation (Mukherjee et al., 2024). Collectively, these oxidative damages impair mitochondrial function. Studies have shown that under hyperoxic conditions, mitochondria in pulmonary epithelial cells exhibit significant structural and functional abnormalities, with a marked decrease in the activity of respiratory chain complexes I and IV, inhibition of oxidative phosphorylation, and alterations in mitochondrial morphology, such as vacuolization and cristae fragmentation (Garcia et al., 2021; Ma et al., 2018). Furthermore, deficiency or mutation of nuclear factor erythroid 2-related factor 2 (Nrf2) has been demonstrated to exacerbate mitochondrial dysfunction and metabolic dysregulation induced by ROS (Cho and Kleeberger, 2020).

Conversely, damaged mitochondria further aggravate OS. Impaired mitochondrial quality control mechanisms lead to the accumulation of dysfunctional mitochondria (Napolitano et al., 2021), disrupted mitochondrial dynamics (e.g., imbalance between fusion and fission) (Ten and Ratner, 2020; Al Ojaimi et al., 2022), and compromised antioxidant defense systems, collectively resulting in sustained ROS elevation, energy deficiency, and eventual activation of apoptosis (Hu and Zhou, 2022; Hu et al., 2022). In neonatal lungs, mitochondria generate more ROS compared to adults (Kindermann et al., 2019), and neonatal animals also show greater tolerance to hyperoxic conditions than mature specimens (Perez et al., 2019). However, preterm infants exposed to non-lethal OS during the critical postnatal intervention window (days 1–14) develop mitochondrial dysfunction characterized by permeability transition pore opening, which fundamentally contributes to arrested alveolar development in BPD pathogenesis (Ten, 2017). The OS burden in BPD can be quantitatively monitored through 8-OHdG levels in airway biofluids, where this DNA damage biomarker not only discriminates BPD infants from healthy controls (p < 0.05) but also exhibits a positive correlation with disease severity scores (r = 0.68, p < 0.05) (Hsiao et al., 2021; Li et al., 2025).

Despite established links between OS and mitochondrial damage, key questions remain. The temporal sequence of events, specifically whether mtDNA damage or ETC dysfunction occurs first, is still debated. Furthermore, while Nrf2 is a promising therapeutic target, its systemic activation must be carefully evaluated due to its pleiotropic roles in other developmental processes. Future research should employ time-resolved models to dissect this causality and develop lung-specific Nrf2 delivery systems.

### 2.2 Immune inflammation

As the most prevalent chronic respiratory disease in preterm infants, BPD is pathologically characterized by a dysregulated inflammatory cascade (Watterberg et al., 1996). Emerging evidence positions mitochondrial dysfunction as a central regulator of pulmonary immune homeostasis disruption in this process. Mechanistically, impaired mitochondria release damage-associated molecular patterns, including mtDNA, mitochondrial ROS (mtROS), ATP, and cardiolipin. These molecules activate pattern recognition receptors, including Toll-like receptors and NLRP3 inflammasome components, thereby triggering pro-inflammatory cytokine cascades (Marzetti et al., 2024; Marchi et al., 2023; Mishra et al., 2021; Chen et al., 2023). Previous studies demonstrate that hyperoxia exacerbates this response through tumor necrosis factor-α (TNF-α) overproduction, which has been shown to depolarize mitochondrial membranes and enhance oxidative-inflammatory interactions in rodent models, potentially establishing a self-perpetuating injury cycle (Mu et al., 2021; Liu et al., 2025). Importantly, this maladaptive immune phenotype correlates directly with BPD severity, while targeted inhibition of specific inflammatory mediators demonstrates therapeutic potential in preclinical models (Lao et al., 2022).

The dual-phase macrophage polarization shift represents a hallmark feature of BPD pathogenesis. Under physiological conditions, M1/M2 macrophages maintain a dynamic equilibrium; however, disease progression is characterized by early M1-dominant pro-inflammatory responses followed by pathological M2 predominance (Mestan et al., 2025; Zhen et al., 2021). Mounting evidence indicates that mtROS-mediated NF-κB activation in macrophages significantly amplifies the inflammatory cascade (Yang et al., 2022). Mesenchymal stem cell-derived exosomes (MSC-exosomes) carrying mitochondrial components, including mt-DNA and oxidative phosphorylation proteins, have emerged as a cell-free therapeutic strategy. Experimental data from lipopolysaccharide (LPS)-induced lung injury models demonstrate that MSC-exosomes restore alveolar macrophage mitochondrial function (increased ATP production, reduced ROS) and polarize macrophages toward an anti-inflammatory phenotype, thereby ameliorating pulmonary inflammation (Xia et al., 2022). Furthermore, studies demonstrate that in hyperoxia-induced BPD models, impaired autophagic signaling exacerbates pulmonary inflammatory injury and promotes disease progression, a process closely linked to dysregulated mitochondrial quality control (Mohsin et al., 2021). Zhang L. et al. (2020) demonstrated through murine and baboon models that impaired autophagic activity exacerbates BPD pathogenesis by disrupting alveolarization, amplifying inflammatory responses, and dysregulating mitophagy, thereby establishing autophagy as a critical mechanism in maintaining lung homeostasis.

Mitochondrial-immune interactions are increasingly recognized as functionally significant in BPD pathogenesis; however, substantial translational challenges persist. A fundamental unresolved issue concerns whether mitochondrial dysfunction acts as a primary initiator or merely an amplifier of pulmonary inflammation. Furthermore, the heavy reliance on hyperoxia and LPS models in current research provides limited insight into the complex immune microenvironment characteristic of human BPD, which typically involves coexisting perinatal insults such as infection and mechanical ventilation. Future investigations should prioritize the development of clinically relevant multi-hit models and rigorously evaluate whether targeted enhancement of mitochondrial function in immune cells can disrupt inflammatory pathways without compromising host defense mechanisms.

### 2.3 Pulmonary vascular and alveolar development abnormalities

Alveolar simplification and abnormal pulmonary vascular development are key features of new BPD (Gilfillan et al., 2021). Accumulating evidence underscores the pivotal role of mitochondrial dysfunction in these processes. In pulmonary endothelial cells, mitochondria drive proliferation via glycolysis and fatty acid oxidation, metabolic pathways whose disruption impairs lung morphogenesis (Yao et al., 2019). Clinically, umbilical vein endothelial cells from neonates with BPD exhibit compromised mitochondrial respiration (reduced oxygen consumption) alongside elevated OS (increased superoxide/H_2_O_2_ production), directly linking endothelial mitochondrial failure to adverse outcomes (Kandasamy et al., 2017).

In alveolar pathogenesis, AT2 cells, which are highly dependent on mitochondrial integrity, require balanced mitochondrial dynamics for surfactant homeostasis. Even transient exposure to hyperoxia triggers persistent mitochondrial dysfunction, characterized by metabolic reprogramming (e.g., impaired oxidative phosphorylation) and structural disorganization, which collectively disrupt alveolar development (Garcia et al., 2021). A mechanistic study published in 2022 demonstrated that hyperoxia suppresses the SMAD3/ERK1/2 signaling pathway, leading to mitochondrial membrane depolarization, excessive fission, and fusion impairment, culminating in AT2 cells apoptosis (Jiang et al., 2022). Chung et al. (Chung et al., 2019) further demonstrated that mitofusin (Mfn) 1/2 deficiency disrupts phospholipid synthesis and exacerbates pulmonary fibrosis. Notably, recent advances implicate lncRNAs in hyperoxia-induced AT2 cells damage: these downregulated lncRNAs modulate Mfn expression, exacerbating mitochondrial dysfunction and apoptotic cascades (Liu et al., 2025).

These evidence underscores mitochondria as a nexus integrating metabolic function and structural development in the lung. A significant gap lies in understanding the interdependence of alveolar and vascular mitochondrial health. Does primary dysfunction in endothelial cells precede and drive AT2 cell injury, or *vice versa*? The answer could dictate the optimal cellular target for therapy. The discovery of lncRNAs regulating mitochondrial dynamics opens a new layer of regulation, but their cell-specific expression patterns and interactions with other signaling networks (e.g., VEGF, Wnt) in BPD are entirely unexplored. The prevailing focus has been on hyperoxia-induced fission; however, the potential role of impaired mitochondrial fusion as a contributor to BPD pathogenesis warrants equal attention.

## 3 Mitochondria-dependent cell death in BPD

**FIGURE 2:**
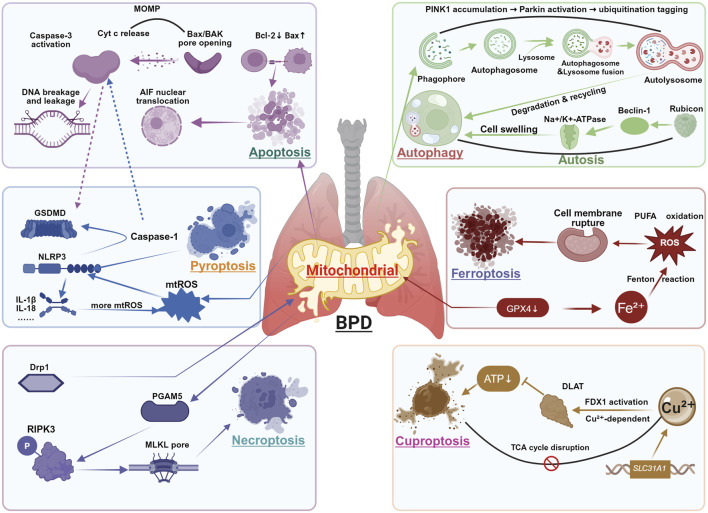
Mitochondria-Dependent Cell Death in BPD. This figure integrates six major cell death modalities regulated by mitochondrial dysfunction in BPD pathogenesis: Apoptosis: Mitochondrial outer membrane permeabilization (MOMP) through Bax/Bak pore formation enables cytochrome c (Cyt c) release, activating caspase-3 and inducing DNA fragmentation. Pyroptosis: Mitochondrial ROS (mtROS) and DNA release activate NLRP3 inflammasome, promoting caspase-1-mediated cleavage of GSDMD and subsequent IL-1β/IL-18 maturation. Necroptosis: TNF-α signaling through RIPK3 phosphorylates MLKL, forming plasma membrane pores independent of caspase activity. Autophagy: PINK1-Parkin mediated mitophagy clears damaged mitochondria through lysosomal degradation, while excessive activation may promote autophagic cell death. Ferroptosis: GPX4 inhibition leads to iron-dependent lipid peroxidation, exacerbated by mitochondrial ROS generation and system Xc-dysfunction. Cuproptosis: Copper accumulation induces DLAT oligomerization and subsequent proteotoxic stress, disrupting mitochondrial TCA cycle function. Key crosstalk mechanisms: ① Caspase-3 cleaves GSDMD to initiate pyroptosis when apoptosis is blocked; ② mtDNA released during apoptosis activates NLRP3 inflammasome; ③ Mitochondrial ROS generation amplifies all death pathways.

In the pathogenesis of BPD, mitochondria act as central hubs coordinating multiple cell death pathways, which collectively contribute to alveolar simplification and impaired vascular maturation. Exposure of the preterm lung to hyperoxia, infection, and mechanical stress induces mitochondrial damage, leading to metabolic disruption, oxidative stress amplification, and inflammatory activation. These dysfunctions converge to activate distinct yet interconnected cell death mechanisms, including apoptosis, pyroptosis, necroptosis, autophagy-dependent death, ferroptosis, and the recently identified cuproptosis. Together, these pathways form a vicious cycle of mitochondrial failure, sustained inflammation, and developmental arrest. In the following sections, we systematically dissect the mitochondrial regulation of each cell death modality in BPD (Figure 2).

### 3.1 Apoptosis

In BPD, apoptosis is primarily mediated through the mitochondrial (intrinsic) pathway, with caspase-3 acting as the key executioner. Notably, caspase-3 also facilitates crosstalk with other cell death modalities, such as pyroptosis (Jiang et al., 2020; Bhat et al., 2023). While the extrinsic pathway can be initiated by exogenous death ligands, this section will focus on the intrinsic pathway due to its central role in mitochondrial dysfunction during BPD pathogenesis.

The intrinsic pathway is characterized by mitochondrial outer membrane permeabilization (MOMP), predominantly mediated by Bax/Bcl2-antagonist killer oligomerization, which forms pores in the outer mitochondrial membrane, promoting the cytoplasmic release of Cyt c and mtDNA (Riley et al., 2018; Victorelli et al., 2023). Released mtDNA activates the cGAS-STING pathway, leading to interferon-beta production and caspase-independent cell death (Wang et al., 2025). Concurrently, MOMP may activate TNF-dependent necrosis and NF-κB signaling through caspase-independent mechanisms, particularly when apoptosis execution is blocked (Giampazolias et al., 2017). MtROS overproduction represents another critical apoptotic mechanism, disrupting mitochondrial ultrastructure and function, leading to apoptosis-inducing factor release, DNA damage, and both caspase-dependent and -independent apoptosis (Yuan et al., 2020).

In BPD pathogenesis, hyperoxia and infection exacerbate mitochondrial dysfunction through these mechanisms. Hyperoxic exposure induces excessive ROS generation in alveolar epithelial mitochondria, causing mtDNA damage, membrane potential collapse, and imbalance of Bcl-2 family proteins—upregulating pro-apoptotic Bax while downregulating anti-apoptotic Bcl-2 expression (Singh et al., 2019; Kaloni et al., 2023). These mitochondrial alterations trigger the release of Cyt c and subsequently activate the caspase-9/caspase-3 cascade (Hu and Zhou, 2022; Carneiro and El-Deiry, 2020). Notably, the Notch4/DLL4 signaling pathway serves as a central regulator in the infection-mediated apoptosis, as evidenced by *E. coli* intrauterine infection models showing upregulated Notch4/DLL4 expression and enhanced pulmonary microvascular endothelial cell apoptosis (Zhan et al., 2021).

Several mitochondrial protective mechanisms are compromised in BPD. SIRT1 deficiency exacerbates mitochondrial dysfunction by promoting p53 acetylation and Bax expression (Singh and Ubaid, 2020; Dong et al., 2021), and clinical observations reveal significantly decreased SIRT1 alongside increased SENP1 expression in peripheral mononuclear cells of BPD preterm infants (Tan et al., 2018). The PI3K/AKT/ETS1 signaling pathway enhances mitochondrial function and suppresses Bax expression, attenuating AT2 cells apoptosis and hyperoxic lung injury (He et al., 2023). Nrf2-mediated antioxidant responses regulate mtROS levels, protecting against hyperoxia-induced oxidative stress and alveolar simplification (Yu and Xiao, 2021; Tamatam et al., 2020), with lncRNAs MALAT1 further modulating Keap1/Nrf2 signaling pathway to mitigate mitochondrial damage (Zhang et al., 2021). Additionally, mitochondrial-localized apoptosis-inducing factor shows reduced expression in BPD infants, suggesting its role in maintaining mitochondrial homeostasis (Zhang et al., 2021). Vitamin D demonstrates therapeutic potential by targeting mitochondrial pathways to reduce apoptosis and inflammation while inhibiting MEK1/2-ERK1/2 signaling pathway to improve alveolarization, as supported by murine studies confirming its ability to restore mitochondrial function, attenuate pulmonary fibrosis, and reduce inflammation (Hu et al., 2022).

While mitochondrial apoptosis is well-established in BPD, its crosstalk with other death pathways remains inadequately explored. Notably, the contextual switch between caspase-3-mediated apoptosis and GSDME-dependent pyroptosis under hyperoxic stress warrants deeper investigation. Furthermore, most current interventions target downstream effectors rather than upstream mitochondrial priming events. Future studies should clarify whether blocking MOMP or modulating Bcl-2 family proteins in a cell-specific manner can more effectively decouple apoptosis from broader inflammatory amplification.

### 3.2 Pyroptosis

In the pathogenesis of BPD, mitochondria-dependent pyroptosis contributes to disease progression through multiple molecular mechanisms. This programmed inflammatory cell death, orchestrated by the caspase superfamily, plays a pivotal role in pulmonary injury via canonical (e.g., caspase-1) or non-canonical pathways (Yu et al., 2021). When mitochondrial dysfunction is induced by stimuli such as hyperoxia or infection, the resulting mtROS promote oxidative modification of GSDMD, which enhances the cleavage efficiency of caspase-1 (Xia et al., 2020). Concurrently, mtDNA release and Ca^2+^ overload leads to further activation of the NLRP3 inflammasome (Murakami et al., 2012; Liu et al., 2018). A recent study demonstrated that hyperoxia exposure markedly increased mtROS levels in neonatal mouse lung tissue, activated the NLRP3 inflammasome as well as promoted GSDMD cleavage dependent on caspase-1 and interleukin-1β release, ultimately resulting in macrophage pyroptosis and impaired alveolar development (Tu et al., 2025). This finding is corroborated by the study of Liao and colleagues (Liao et al., 2015), who demonstrated that NLRP3 gene knockout mice exhibited markedly attenuated inflammatory responses and reduced alveolar simplification under hyperoxic conditions. Notably, oxidized phosphatidylcholine, a product of phospholipid peroxidation, was found to induce mtROS accumulation, thereby amplifying the positive feedback loop for NLRP3 inflammasome activation (Yeon et al., 2017).

Regarding therapeutic interventions, multiple studies have demonstrated the potential of targeting this pathway. Caffeine inhibits both the NLRP3 inflammasome and NF-κB signaling, thereby reducing hyperoxia-induced pro-inflammatory cytokine production and improving alveolar development (Chen et al., 2020). Furthermore, 1″-Acetoxychavicol acetate, a naturally occurring phenylpropanoid compound extracted from the rhizomes of Alpinia species, attenuates pyroptosis and hyperoxia-induced pulmonary injury by inhibiting mtROS production and the release of oxidized mtDNA, thereby blocking NLRP3 inflammasome activation (Sok et al., 2021). Studies in preterm baboon models have also demonstrated that interleukin-1 receptor antagonists alleviate pulmonary inflammation and fibrosis, further substantiating the pathological relevance of this pathway (Dapaah-Siakwan et al., 2019).

The non-canonical pyroptosis mechanism operates through caspase-4/5/11, which directly cleaves GSDMD to initiate pyroptosis (Weir and Vince, 2022). However, its specific role in BPD requires further elucidation. Of particular interest, dysregulated iron metabolism plays a distinct role in BPD-associated pyroptosis. Iron-activated ROS can promote pyroptosis via the translocase of outer mitochondrial membrane 20-Bax-caspase-GSDME pathway (Wu et al., 2023), providing novel insights into the association between iron overload in preterm infants and BPD. Furthermore, the temporal regulation of pyroptosis, specifically whether it occurs predominantly during the early inflammatory exacerbation phase or the late fibrotic remodeling stage, remains unclear and merits further investigation.

### 3.3 Necroptosis

Necroptosis is a regulated form of cell death characterized by plasma membrane rupture and release of damage-associated molecular patterns, which trigger robust inflammatory cascades (Grootjans et al., 2017; Faust and Mangalmurti, 2020). This process can be initiated by diverse stimuli, including oxidative stress, infection, and cytokine signaling (Vanlangenakker et al., 2012).

In BPD, necroptosis serves as a cell death pathway with significant implications. Earlier studies demonstrated that mitochondria were not essential for necroptosis, as forced RIPK3 activation could induce necroptosis even in mitochondria-deficient cells (Tait et al., 2013). However, accumulating evidence now reveals a strong connection between mitochondrial dysfunction and necroptotic pathways. In hyperoxia-induced models, RIPK3 not only boosts bioenergetics and mtROS production via upregulation of glycogen phosphorylase and pyruvate dehydrogenase complex E1α subunit, but also elevates mitochondrial NADPH oxidase 4 levels through post-transcriptional mechanisms, directly inducing mitochondrial damage (Uni and Choi, 2022). These mtROS further facilitate RIPK1 autophosphorylation and RIPK3 recruitment, establishing a positive feedback loop (Schenk and Fulda, 2015). Notably, the necrosome of RIPK1/RIPK3/MLKL can migrate to mitochondrial membranes, activating PGAM5 to disrupt mitochondrial dynamics and glutathione metabolism, thereby exacerbating oxidative stress (Cheng et al., 2021; She et al., 2019).

During BPD progression, hyperoxia exposure activates necroptotic pathways through multiple mechanisms. By driving mTOR-dependent mitochondrial fission, TREM-1 triggers macrophage necroptosis, which in turn aggravates acute lung injury. This pathway involves the phosphorylation of Drp1 and aberrant activation of mitophagy (Zhong et al., 2023). Concurrently, the CCR5 signaling pathway upregulates RIPK3 expression via NF-κB activation, promoting macrophage accumulation in lung tissues and pro-inflammatory cytokine release (Chen et al., 2021). Clinical data reveal significantly elevated pro-inflammatory cytokine concentrations in bronchoalveolar lavage fluid from BPD infants (Köksal et al., 2012). These cytokines act both as necroptosis inducers and downstream effectors by impairing mitochondrial function, creating a vicious cycle. Recent studies highlight that NLRP3/caspase-1 axis activation not only induces GSDMD-mediated pyroptosis but also facilitates GSDMD pore formation in mitochondrial membranes, driving ROS release and subsequent RIPK3/MLKL-dependent necroptosis (Liu et al., 2016; Ehrhardt et al., 2016). These findings provide novel insights into BPD pathogenesis and suggest that targeting key necroptotic molecules may represent a promising therapeutic strategy.

Although the involvement of mitochondria in necroptosis was initially controversial, this issue has now been sufficiently addressed and clarified. Nevertheless, key unanswered questions remain, including whether necroptosis preferentially affects endothelial or epithelial compartments and how it interacts with concurrent apoptosis or pyroptosis. The potential spatial association of necrosomes with mitochondrial membranes also suggests organelle-specific signaling hubs that could be disrupted therapeutically.

### 3.4 Autophagy

Autophagy, an evolutionarily conserved catabolic process, plays a pivotal role in maintaining cellular homeostasis by clearing damaged organelles and proteins via lysosomal degradation (Racanelli et al., 2018; Klionsky et al., 2021). While primarily a survival mechanism, dysregulated autophagy can contribute to programmed cell death. This includes autophagy-dependent cell death (ADCD), which encompasses subtypes such as mitophagy and autosis (Galluzzi et al., 2018; Jung et al., 2020). Studies demonstrate that autophagy participates in multiple stages of pulmonary development, particularly during rapid fetal and neonatal growth phases, where it regulates the turnover and energy metabolism of AT2 cells (Yeganeh et al., 2019). In the pathogenesis of BPD, dysregulated autophagy is recognized as a critical factor. Moderate autophagy maintains AT2 cells homeostasis and promotes alveolar repair, whereas excessive or impaired autophagy exacerbates cellular apoptosis and pulmonary tissue injury, ultimately contributing to BPD progression (Zhang et al., 2018).

Autosis, a novel ADCD modality regulated by Na^+^/K^+^-ATPase, is characterized morphologically by pathological deposition of autophagosomes/autolysosomes, nuclear convolution, and late-stage perinuclear space dilation (Liu et al., 2013; Liu et al., 2023). The classification of autosis as ADCD remains debated because the death process cannot be fully reversed by autophagy inhibition alone. However, current evidence indicates that autosis is closely associated with dynamic autophagic flux alterations, specifically early-stage autophagy enhancement coupled with late-stage autophagy inhibition. This biphasic regulation, mediated by rubicon (a Beclin 1-interacting protein), collectively promotes autosis (Nah et al., 2022). Notably, autosis initiation requires direct binding between Na^+^/K^+^-ATPase and the autophagy-related protein Beclin1, an interaction activated under ischemic stress that disrupts ion homeostasis and potentially induces mitochondrial dysfunction (Fernández et al., 2020). At the subcellular level, autosis involves abnormalities in multiple organelles (endoplasmic reticulum, mitochondria, nuclear membrane), with mitochondrial impairment potentially exacerbating cell death through energy metabolism dysregulation (Nah et al., 2020a). While the direct relationship between autosis and BPD remains unclear, the autophagy-mitochondrial axis warrants attention: (1) Autophagy dysregulation (e.g., via Rubicon-Beclin1-Na^+^/K^+^-ATPase pathway disruption): may promote cell death by impairing mitochondrial quality control (Li Y. et al., 2020); (2) BPD-associated stressors (e.g., hypoxia/reperfusion) can induce autophagy-dependent death (Nah et al., 2020b), suggesting analogous mechanisms may contribute to mitochondrial dysfunction in BPD. Future studies should elucidate the molecular interplay between autophagy and mitochondria during autosis to clarify its pathological role in BPD.

In contrast, mitophagy demonstrates a more direct mechanistic association in BPD research. Mitophagy refers to the selective removal of damaged mitochondria via autophagy, thereby maintaining a dynamic balance of mitochondrial quantity and quality (Onishi et al., 2021). Under normal development or mild stress conditions, mitochondria maintain functional integrity through fusion and fission processes (Pickles et al., 2018; Chan, 2020). Proteins such as Drp1, Mfn1/2, and optic atrophy 1 work in concert to regulate mitochondrial dynamics, ensuring the removal of damaged mitochondria while preserving oxidative phosphorylation efficiency (Tong et al., 2020; Deng Z. et al., 2024; Zhang et al., 2019). However, in pathological states such as BPD and other lung injuries, mitochondrial fragmentation increases, and membrane depolarization induces PINK1 accumulation at the outer mitochondrial membrane, which recruits and activates Parkin. Subsequently, ubiquitination and MAP1A/1B-LC3 binding facilitate the autophagic clearance of mitochondria (Kandasamy et al., 2023; Araya et al., 2019). Moreover, FUN14 domain-containing protein 1, a mitophagy receptor under hypoxic conditions, participates in the recognition and clearance of mitochondria by interacting with LC3 (Liu et al., 2022). Multiple studies have confirmed that the PINK1-Parkin pathway plays a dual role in BPD models: On one hand, in LPS-induced acute lung injury models, the PINK1-Parkin pathway is activated, accompanied by mitochondrial membrane potential loss, upregulation of Bcl-2-associated agonist of cell death protein, and downregulation of Bcl-2, suggesting cross-regulation between mitophagy and apoptosis (Zhang Z. et al., 2020). On the other hand, in hyperoxia-exposed neonatal rat AT2 cells, the upregulation of PINK1, Parkin, and NIX suggests that the buildup of dysfunctional mitochondria may be one of the core factors in the pathological process of BPD (Yu et al., 2020). Lipoxin A4 has been shown to mitigate oxidative stress and airway inflammation by inhibiting this pathway, thereby alleviating BPD-related lung injury (Wu et al., 2019).

While mitophagy serves as a critical quality control mechanism by eliminating mitochondria impaired by OS and maintaining alveolar development, extensive evidence shows that excessive activation (such as through the CerS1/Drp1-dependent ceramide pathway) or functional defects (such as lysosomal metabolic abnormalities mediated by GBA1) can induce cell death across various disease conditions (Oleinik et al., 2023; Oleinik et al., 2025; Dasari et al., 2017). This duality underscores a broader translational challenge inherent to autophagy as a whole: although mechanistically compelling pathways such as Rubicon-regulated autosis and PINK1–Parkin-mediated mitophagy have been delineated, their net outcomes are highly context-dependent, influenced by stress intensity, timing, and cellular milieu. A crucial unresolved issue lies in deciphering how autophagic flux is spatiotemporally regulated across distinct cell types in the developing lung. We hypothesize that strategically enhancing early mitophagy may promote clearance of dysfunctional organelles and attenuate inflammation, whereas curbing aberrant autosis could safeguard ion homeostasis and metabolic integrity. Future efforts should prioritize elucidating the regulatory networks of mitophagy within BPD, particularly its crosstalk with mitochondrial biogenesis and ER-mitochondria communication, to guide targeted therapeutic interventions.

### 3.5 Ferroptosis

In the pathogenesis of BPD, ferroptosis, driven by iron dependence and lipid peroxide/ROS accumulation, demonstrates close association with mitochondrial dysfunction (Jiang et al., 2021). The hallmark morphological features of ferroptosis, including mitochondrial shrinkage, outer mitochondrial membrane rupture, and reduced mitochondrial cristae (Li J. et al., 2020), have been observed in hyperoxia-induced BPD models (Jia et al., 2021). Furthermore, cumulative iron supplementation methods, including blood transfusion, have been identified as independent risk factors for BPD development (Garcia et al., 2023; Bahr et al., 2024), suggesting that iron metabolism dysregulation may critically contribute to BPD pathogenesis.

The initiation involving ferroptosis two major pathways: the extrinsic pathway, mediated by the system Xc-transporter system, and the intrinsic pathway, linked to the activity of GPX4 (Zhang et al., 2023). Mitochondria, serving as the primary intracellular iron storage sites, contain approximately 20%–50% of the total cellular iron content (Chipuk et al., 2021; Jhurry et al., 2012). Mitochondrial iron metabolism is primarily regulated by mitoferrin and voltage-dependent anion channels (Ben Zichri-David et al., 2025). Under conditions of mitochondrial iron overload, Fe^2+^ generates excessive ROS via the Fenton reaction, attacking mitochondria membranes rich in polyunsaturated fatty acids and triggering a lipid peroxidation cascade (Lv et al., 2023; Veeckmans et al., 2024). Under cystine deprivation, accelerated glutaminolysis drives the tricarboxylic acid (TCA) cycle, leading to mitochondrial membrane potential hyperpolarization with consequent excessive ROS production (Gao et al., 2019). This process may be further amplified in the hyperoxic environment characteristic of BPD.

The Nrf2 signaling pathway exerts protective effects against mitochondrial oxidative damage by regulating the expression of ferroptosis-related proteins, including GPX4 and SLC7A11 (Deng J. et al., 2024). Activation of the Keap1-Nrf2/HO-1 pathway has been shown to mitigate ferroptosis in LPS-induced acute lung injury, ameliorating alveolar inflammation and oxidative stress (Li et al., 2021), thereby providing novel therapeutic insights for BPD management. Animal studies have confirmed that hyperoxia exposure induces ferroptosis prior to impaired lung development (Chou and Chen, 2022), suggesting a critical role of ferroptosis in hyperoxia-induced lung injury. Meanwhile, recent studies (Mao et al., 2021; Deng H. et al., 2025; Deng R. et al., 2025) have identified that mitochondrial enzymes, including DHODH, LDHB, and MCI, suppress ferroptosis by reducing mitochondrial lipid peroxides through their respective mechanisms of promoting the reduction of CoQ to CoQH2. Therefore, targeted modulation of mitochondrial-dependent ferroptosis may represent a promising therapeutic strategy for BPD intervention.

Although ferroptosis is morphologically and biochemically distinct, its regulators (e.g., GPX4, Nrf2, DHODH) overlap with those in other oxidative death pathways, raising questions about its uniqueness in BPD. The role of mitochondrial iron sequestration and its interaction with Fenton chemistry requires further clarification in lung development. Importantly, most evidence comes from cancer models; rigorous validation in BPD-relevant systems is essential. We propose that ferroptosis may be especially relevant in infants receiving transfusions or parenteral nutrition, linking iatrogenic iron overload to poor outcomes.

### 3.6 Cuproptosis

Cuproptosis is a novel copper-dependent form of cell death, with its core mechanism involving the abnormal accumulation of copper ions within the mitochondria and their interaction with key metabolic proteins (Liu et al., 2024). Cell death mediated by copper ion carriers, such as elesclomol, is not blocked by inhibitors of apoptosis, ferroptosis, or necroptosis, but can be inhibited by glutathione through the chelation of copper ions, suggesting its unique death pathway. Notably, cells that rely on mitochondrial respiration are significantly more sensitive to cuproptosis than glycolysis-dependent cells, and inhibitors of the mitochondrial electron transport chain can weaken the effects of cuproptosis, highlighting the special role of mitochondrial metabolism in this process (svetkov et al., 2019; Tsvetkov et al., 2022).

At the molecular level, DLAT, which is a key enzyme of the TCA cycle, directly binds copper ions, leading to promoted lipoylation modification and subsequent protein aggregation (Tsvetkov et al., 2022). Additionally, ferrochelatase 1 has been identified as a key effector molecule in cuproptosis, driving the cell death proce ss by regulating protein acylation modifications as well as degrading iron-sulfur cluster proteins (Dreishpoon et al., 2023). In acute lung injury, hyperoxic conditions may lead to the upregulation of copper transporter *SLC31A1* expression, resulting in mitochondrial copper overload, which subsequently activates the cuproptosis pathway (Yu et al., 2025). This copper-dependent mitochondrial dysfunction may be related to oxidative stress and abnormal alveolar development in BPD. However, only two cuproptosis-associated genes (*NFE2L2* and *GLS*) and a five-gene signature (*NFATC3*, *ERMN*, *PLA2G4A*, *MTMR9LP*, *LOC440700*) have been preliminarily identified for BPD (Jia et al., 2023), with further exploration of its mechanisms still needed.

As the most recently identified form of cell death, cuproptosis still lacks strong *in vivo* evidence in the context of BPD. Given that it depends on mitochondrial respiration, this process is especially relevant in developing lungs, which experience significant metabolic transitions. Major unanswered questions involve which cell types are most susceptible to copper induced toxicity, how hyperoxia affects the expression of copper transporters and the accumulation of mitochondrial copper, and whether genetic variations in copper metabolism or antioxidant related genes contribute to differences in disease presentation.

## 4 Conclusion and perspectives

Mitochondria unequivocally stand as the central hub orchestrating disparate cell death pathways in BPD. Although not all types of cell death directly depend on mitochondria, accumulating research demonstrates the multifaceted involvement of mitochondria in cell death pathways. In addition to the cell death modalities mentioned in this review, mitochondria act as direct drivers in multiple contexts: in mitochondrial permeability transition-driven necrosis, the opening of the mitochondrial permeability transition pore leads to loss of membrane potential and energetic collapse, serving as a direct trigger for cell death (Tsujimoto et al., 2006); in extrinsic apoptosis, although initiation relies on membrane signaling via death receptors (e.g., Fas/TNF receptor 1), mitochondria participate in death signal amplification through caspase-8-mediated BH3-interacting domain death agonist cleavage, inducing MOMP (Tummers and Green, 2017); in lysosome-dependent cell death, mitochondrial functional regulation (e.g., ROS production and calcium flux) is believed to synergistically modulate lysosomal membrane permeabilization and cathepsin release (Milani et al., 2023). The intricate interplay of apoptosis, pyroptosis, necroptosis, ferroptosis, cuproptosis, and dysregulated autophagy within the mitochondrial landscape underscores its role as a unifying platform that integrates diverse death signals. As mitochondrial functions in cellular stress response, redox balance, and inflammatory signaling continue to be uncovered, their role in cell fate regulation has become increasingly prominent. This functional versatility solidifies the organelle’s position as the central regulator of cell death. Mitochondria are no longer merely “powerhouses” but indispensable “command hubs” and “executors” within the cell death network, coordinating multiple demise programs through their central positioning in metabolism, signaling, and organelle interactions.

Translating these mechanistic insights into clinical applications is the next critical frontier. Promising mitochondria-derived biomarkers include cell-free mtDNA and 8-OHdG in tracheal aspirates or blood, which correlate with disease severity and could enable early identification of high-risk infants (Li et al., 2025; Fernandez-Gonzalez et al., 2025). Additionally, specific signatures of inflammation (e.g., NLRP3 activity, IL-1β) and iron overload (e.g., non-transferrin bound iron) may further stratify risk and guide personalized interventions.

Therapeutically, targeting the mitochondrial hub offers a strategic advantage over targeting individual pathways. Potential therapeutic strategies comprise enhancing mitochondrial quality through compounds such as MitoQ to scavenge mtROS or Urolithin A to promote mitophagy (Yan et al., 2023; Mohsin et al., 2025); modulating cell death execution via NLRP3 inflammasome inhibitors (e.g., Anakinra, though requiring careful timing) (Green et al., 2022), ferroptosis inhibitors (e.g., Liproxstatin-1) (Bao et al., 2022), or necroptosis blockers (e.g., RIPK1 inhibitors); boosting endogenous protection by activating the Nrf2 antioxidant pathway with agents like Sulforaphane or using Melatonin to support mitochondrial function (Cho et al., 2019; Wang and Gao, 2021); and developing cell-based therapies that leverage the mitochondrial transfer capability of MSC-exosomes to repair damaged lung cells.

Given the current scarcity of direct research data on BPD, discussions of certain cell death modalities in this review rely on evidence from other pulmonary diseases, particularly regarding whether specific types of cell death contribute to immature lung cell injury under hyperoxia or infection—their precise role remains unclear. It should be noted that cell death induced by acute lung injury is a critical trigger for pulmonary developmental disruption, and such developmental abnormalities constitute a core driving force in BPD pathogenesis. Current BPD research is largely confined to cellular experiments and animal models, predominantly employing hyperoxia or pathogen-associated stimuli to simulate BPD pathological processes. However, these single-insult models struggle to fully replicate the complex, multifactorial clinical reality of BPD.

Oxidative stress, as a key contributing factor in BPD pathogenesis, can activate multiple programmed cell death pathways, thereby damaging pulmonary epithelial and endothelial cells. Nevertheless, oxidative stress constitutes merely one facet of the pathological spectrum in BPD, while the mechanisms that genuinely reflect disease complexity are substantially more extensive. Currently, there is a lack of direct, robust preclinical evidence supporting the safety and efficacy of targeted interventions for BPD treatment. Excessive or non-selective inhibition of cell death programs may interfere with normal developmental processes and cause irreversible adverse effects, making it particularly critical to define therapeutic windows and validate the safety of target mechanisms.

Encouragingly, in recent years, significant advances have been made in theoretical research on BPD pathogenesis. A deeper elucidation of the organelle-level mechanisms regulating immature lung cells and the identification of different cell death pathways will open new avenues for targeted BPD therapies. Additionally, adopting precision phenotyping strategies to predict infants likely to benefit from intervention will further enhance these therapeutic approaches. With continuous advancements in technology and enhanced interdisciplinary integration, research on this mitochondrial-centric network of cell death regulation holds promise for delivering more prospective and clinically actionable treatment strategies for BPD.

## Search strategy

A comprehensive literature search was conducted using the electronic databases PubMed, Web of Science, and Embase from inception to August 2024. The search strategy employed a combination of keywords and MeSH terms including, but not limited to: “bronchopulmonary dysplasia”, “BPD”, “mitochondria”, “mitochondrial dysfunction”, “oxidative stress”, “inflammation”, “cell death”, “apoptosis”, “autophagy”, “pyroptosis”, “necroptosis”, “ferroptosis”, and “cuproptosis”. Boolean operators (AND, OR) were used to combine terms. The search was limited to articles published in English. Both preclinical (animal and cell culture) studies and clinical studies were included. The reference lists of retrieved articles were also manually screened to identify additional relevant publications. The final selection of literature was focused on seminal works and the most recent advances elucidating the role of mitochondria and cell death in BPD pathogenesis.
